# Fractures of the Pelvis and Acetabulum: Novel Insights into Diagnostics and Treatment

**DOI:** 10.3390/jcm13237215

**Published:** 2024-11-27

**Authors:** Florian Baumann, Anne Herrmann-Johns, Volker Alt, Viola Freigang

**Affiliations:** 1 Department of Trauma Surgery, University Medical Centre Regensburg, Franz-Josef-Strauss-Allee 11, 93053 Regensburg, Germanyviola.freigang@ukr.de (V.F.); 2 Department for Epidemiology and Preventive Medicine, Medical Sociology, University Regensburg, 93053 Regensburg, Germany; 3 Faculty of Interdisciplinary Studies, Landshut University of Applied Sciences, Am Lurzenhof 1, 84036 Landshut, Germany

Over the past decade, the number of fractures of the pelvis and acetabulum has increased dramatically, by up to 58% [1]. Demographic changes in industrialized societies are leading to socio-economic challenges due to changes in healthcare requirements. Whereas high-energy trauma injuries to the pelvis are regressive, insufficiency fractures among elderly women represent not only a medical issue but also a major economic burden for healthcare systems [2].

The focus of this Special Issue is the exploration of current innovations in the diagnosis and treatment of these injuries. We are pleased to present five excellent publications showcasing the current trends toward minimal invasive surgery and patient-centered medicine in pelvic surgery.

Recently, a large epidemiologic study on fractures showed that the fastest-growing numbers can be seen in acetabular fractures [1]. Innovations in the management of acetabulum fractures could prove to be vital in improving the standard of care. The study by Einhorn et al. [3] compared percutaneous fixation in acetabular fractures to a standard open reduction and internal fixation (ORIF) procedure in terms of the peri-operative data, the long-term outcomes, and the need for conversion to total hip arthroplasty. They found no difference in long-term outcomes; however, the operative time and blood loss were significantly lower in the percutaneous group.

The evaluation of risk factors for post-traumatic osteoarthritis after acetabular fracture was the focus of the study by Wójcicki et al. [4]. They found a trend toward higher rates of secondary arthroplasty dependent on the approach and the time from injury to ORIF. 

Regenbogen et al. [5] also investigated the timing of surgery in acetabular fractures. The authors found no increase in complications in early surgery for geriatric acetabular fractures in their registry-based study. In contrast to younger patients, where early surgery was associated with increased blood loss, the risk of bleeding in geriatric patients was independent of the timing of surgery.

Innovations in digital imaging and 3D technology have transformed preoperative planning and intraoperative guidance in the surgical treatment of pelvic fractures. The studies on pelvic fracture treatment in this Special Issue address two main issues of sacral screw fixation (SSF) for posterior pelvic fractures: (1) potential hyper-compression and (2) functional outcomes after the transfixation of the sacroiliac (SI) joint. 

Three-dimensional visualization may provide improved insights in the evaluation of post-operative radio-morphometry and bone volume after sacral screw fixation (SSF), as shown by the study group [6]. This study revealed that sacral compression occurs only in cases without anterior pelvic ring reduction and fixation. 

The study by Jaeckle et al. [7] investigated gait and joint motion after SSF in pelvic fracture patients. The authors found reduced forward and upward propulsion after SSF, combined with limitations in SI joint mobility, leading to increased hip joint motion demand. This should be considered in post-operative protocols after SSF and in potential indications for implant removal after SFF.

The studies in this Special Issue provide new insights into recent innovations in diagnosing and treating pelvic and acetabulum fractures. However, further research on the socio-economic and psychosocial consequences of fragility fractures of the pelvis and the acetabulum. Despite there being established guidelines for the treatment of osteoporosis, many patients, especially females, do not receive care according to best-practice recommendations [8]. The reasons for this treatment gap include a lack of specialized infrastructure, insufficient interdisciplinary care, and gaps in care continuity [9]. This results in suboptimal treatment, increasing the risk of adjacent fractures and reduced quality of life. Beyond medical and caregiving challenges, the economic dimension of osteoporotic fractures is considerable [10]. Economic analyses have revealed the substantial financial burden that osteoporosis-related fractures place on the German healthcare system. However, diagnosis and healthcare costs in Germany do not reflect the necessity for improvements in treatment pathways for fragility fractures. Despite the increasing incidence of osteoporosis and osteoporotic fractures, the number of diagnoses of osteoporosis and related fractures has decreased over the past ten years. The German healthcare cost per citizen related to osteoporosis was stable from 2015 to 2020. Since these issues are most likely associated with a lack of awareness, further studies are required, highlighting the necessity for improvements in the prevention and treatment of fragility fractures of the pelvis and acetabulum.

In conclusion, the rising incidence of osteoporotic fractures of the pelvis and acetabulum has medical and economic implications. Advancements in surgical techniques and post-operative evaluation are essential in improving patient-centered treatment outcomes. However, we also need to improve interdisciplinary prevention strategies to minimize the socio-economic implications of fragility fractures. 
